# Ultrasound-guided continuous radiofrequency of the C2 dorsal root ganglion for menstrually related migraine: a comparative clinical study

**DOI:** 10.3389/fneur.2026.1807846

**Published:** 2026-09-18

**Authors:** Heng Huang, Liqiang Yang, Jing Liang, Weigang Zhou, Liangliang He

**Affiliations:** 1 Department of Anesthesiology, Hanzhong Central Hospital, Hanzhong, Shaanxi, China; 2 Department of Pain Management, Xuanwu Hospital Capital Medical University, National Medical Center for Neurological Diseases, National Clinical Research Center for Geriatric Diseases, Beijing, China; 3 First Operating Theatre, Hanzhong Central Hospital, Hanzhong, Shaanxi, China

**Keywords:** menstrually related migraine, C2 dorsal root ganglion, continuous radiofrequency, ultrasound guidance, zolmitriptan

## Abstract

**Background:**

Menstrually related migraine (MRM) is a common migraine subtype in women and is often accompanied by gastrointestinal symptoms, anxiety, and depression, leading to substantial functional impairment. Conventional pharmacological treatments show limited efficacy in improving long-term symptoms and quality of life. This study aimed to compare the clinical effectiveness of ultrasound-guided continuous radiofrequency (CRF) of the C2 dorsal root ganglion with conventional zolmitriptan therapy in patients with MRM.

**Methods:**

This retrospective comparative observational study (using prospectively collected clinical data) included 69 patients with MRM treated between January and December 2024. Patients were assigned to a conventional medication group receiving oral zolmitriptan (Group C, *n* = 34) or an ultrasound-guided C2 dorsal root ganglion CRF group (Group R, *n* = 35) based on clinical decision-making. Headache intensity (NRS), attack frequency and duration, nausea and vomiting (VRS), anxiety (HAMA), depression (HAMD), and migraine-related disability (MIDAS) were assessed at baseline and up to 6 months post-treatment. Treatment efficacy and adverse events were assessed at baseline and up to 6 months post-treatment.

**Results:**

Both groups showed significant improvements in headache symptoms and functional outcomes compared with baseline (*p* < 0.001). In the CRF group, significant improvements were observed across all outcome measures from baseline to 6 months, including NRS pain scores (7.5 ± 0.9 to 3.6 ± 0.7), attack frequency and duration, anxiety, depression, and disability scores (all *p* < 0.001). Between-group comparison showed greater improvements in Group R compared with Group C across multiple time points; however, this comparison should be interpreted with caution given the non-guideline-concordant zolmitriptan regimen.

**Conclusion:**

In this retrospective comparative observational study, ultrasound-guided continuous radiofrequency of the C2 dorsal root ganglion was associated with greater improvements in headache symptoms, psychological status, and functional disability compared with oral zolmitriptan therapy in patients with MRM, with a favorable safety profile. These findings should be interpreted with caution given the different treatment modalities and study design limitations.

**Trial registration:**

This study was registered in the Chinese Clinical Trial Registry (ChiCTR2500095771).

## 1 Introduction

Migraine is one of the most common primary headache disorders in clinical practice. Its prevalence in women is roughly two to three times higher than in men, with around 60% of female patients experiencing menstrual migraine (MM), which is closely linked to the menstrual cycle (1, 2). According to the International Classification of Headache Disorders, 3rd edition (ICHD-3) (3), migraine is divided into two subtypes: Pure menstrual migraine (PMM) involves attacks that happen only during menstruation, whereas menstrually-related migraine (MRM) includes attacks during menstruation and at other times cycle.

The decision to focus on MRM rather than general episodic migraine was based on several clinical considerations: (1) MRM represents a distinct migraine subtype with unique pathophysiological features, particularly the interaction between hormonal fluctuations and trigeminovascular activation; (2) MRM is often more refractory to conventional pharmacological treatment than non-menstrual migraine, with patients reporting higher attack frequency, greater severity, and poorer treatment response; (3) MRM is highly prevalent, affecting approximately 60% of women with migraine, and represents a significant unmet clinical need; and (4) the relatively predictable timing of menstrual attacks provides a unique window for evaluating preventive interventions. Furthermore, emerging evidence suggests that cervicogenic factors may play a particularly important role in MRM, making it a clinically relevant population for evaluating C2 DRG-targeted interventions.

In China, the overall prevalence of migraine is about 9.3%, although comprehensive nationwide data on MM are still lacking (4). Patients with MRM often experience nausea and vomiting as accompanying symptoms (5, 6), along with psychological issues such as anxiety and depression (7). These symptoms significantly impact quality of life, making MRM highly disabling and hard to manage (8). The management of MRM primarily encompasses acute treatment, short-term preventive treatment, and long-term preventive treatment (9). Regardless of the treatment phase, pharmacological therapies remain the mainstay, including non-steroidal anti-inflammatory drugs (NSAIDs), calcitonin gene-related peptide (CGRP)-targeted novel drugs, and triptans (9). Among these agents, triptans—including zolmitriptan—are first-line acute abortive treatments for migraine attacks (10). Zolmitriptan, a selective 5-HT1B/1D receptor agonist, is effective for acute treatment of migraine attacks, including those associated with MRM (11). For short-term perimenstrual prophylaxis, triptans may be used in a scheduled, short-course manner (typically 5–7 days perimenstrually) in some clinical guidelines, but they are not recommended for continuous long-term daily prophylaxis due to the risk of medication overuse headache (11). Similar pre-post study designs have been used in recent evaluations of interventional treatments for headache disorders, providing valuable real-world clinical data (12).

Radiofrequency (RF) therapy, as a minimally invasive neuromodulatory technique, has been increasingly applied in chronic pain management. For migraine, emerging evidence supports that RF modulation of specific neural targets can interrupt abnormal pain signaling (13–15). The C2 dorsal root ganglion (C2 DRG) has been identified as an appropriate target for ongoing radiofrequency therapy to treat cervicogenic headache (14, 15). It should be noted that CRF and zolmitriptan represent different treatment modalities—CRF serves as a neuromodulatory intervention with potential preventive effects, while zolmitriptan is primarily an acute abortive treatment. The present study compared these two commonly used clinical approaches in real-world practice, not as competing alternatives, but to provide observational data on their respective clinical outcomes in MRM management.

## 2 Materials and methods

### 2.1 General information

This study received approval from the Ethics Committee of Xuanwu Hospital, Capital Medical University (Approval No.: 2023–212-002, Clinical Study Approval). A retrospective comparative observational study was conducted involving 69 patients with menstrually-related migraine (MRM) who received treatment at Xuanwu Hospital from January to December 2024. All data were retrieved from the Standardized Electronic Medical Record Registration System of Xuanwu Hospital, which records patient information prospectively during clinical encounters. This partially mitigates recall bias but does not eliminate selection bias inherent in non-randomized treatment allocation. Patients were allocated to treatment groups based on clinical evaluation, patient preference, and presence of contraindications, reflecting real-world clinical practice patterns rather than investigator-directed randomization. The two groups received different therapeutic modalities—a neuromodulatory procedure (CRF) versus pharmacological therapy (zolmitriptan)—which were compared observationally. This design does not constitute a traditional cohort study (which follows exposure-defined groups over time) nor a case–control study (which compares cases and controls by outcome status). Rather, it represents a comparative observational analysis of two treatment groups from clinical practice. Treatment allocation was based on patients’ clinical evaluation results, treatment willingness, and presence of drug use contraindications: patients who voluntarily chose oral zolmitriptan therapy and had no relevant drug contraindications were included in the conventional medication group (Group C); those with drug use contraindications, poor response to previous drug therapy, or who voluntarily opted for minimally invasive interventional treatment were included in the ultrasound-guided C2 dorsal root ganglion continuous radiofrequency treatment group (Group R) (Figure 1).

**Figure 1:**
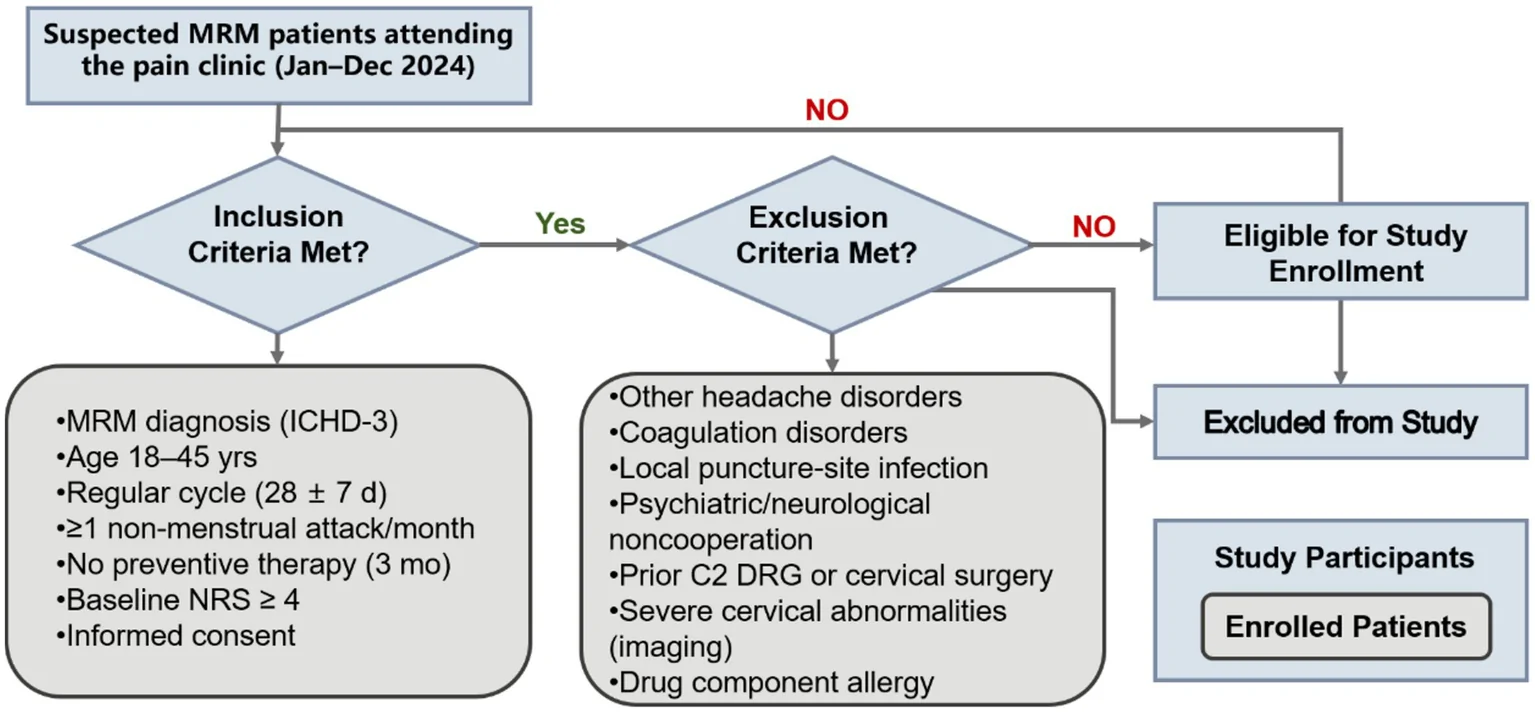
Flowchart of patient screening and enrollment.

Inclusion criteria include: (1) meeting the diagnostic criteria for menstrually related migraine as per the International Classification of Headache Disorders, 3rd edition (ICHD-3) (3); (2) aged 18 to 45 years; (3) having regular menstrual cycles (28 ± 7 days); (4) experiencing at least one migraine attack outside the menstrual period each month; (5) not using preventive migraine medication in the past 3 months; (6) having a Numerical Rating Scale (NRS) score of 4 or higher before treatment; and (7) providing signed informed consent, either personally or from a close family member.

Exclusion criteria include: (1) presence of other headache types; (2) coagulation disorders; (3) local infection at the puncture site; (4) psychiatric or neurological conditions that hinder cooperation during the procedure; (5) history of minimally invasive procedures on the C2 dorsal root ganglion or other cervical spine surgeries; (6) severe cervical spine abnormalities seen on imaging; (7) known allergy to the medication components.

Withdrawal criteria: (1) Severe adverse reactions occurring during treatment, inability to tolerate the treatment; (2) Loss to follow-up or failure to complete the follow-up; (3) Receiving other migraine treatment regimens during the follow-up period. Patient screening flowchart (see Figure 1).

### 2.2 Treatment methods

In this retrospective observational study, treatment selection was determined by the treating physicians and patients in routine clinical practice, not by study investigators.

1. Patients who were prescribed oral zolmitriptan therapy by their treating physicians and had no relevant drug contraindications comprised the conventional medication group (Group C).
2. Patients who underwent C2 DRG CRF treatment—either due to drug contraindications, poor response to previous pharmacological therapy, or patient preference for interventional treatment—comprised the CRF group (Group R). This allocation reflects real-world clinical decision-making rather than investigator-directed study assignment.

#### 2.2.1 Conventional medication group

Patients in the conventional medication group received oral zolmitriptan tablets (2.5 mg per dose) as prescribed by their treating physicians in routine clinical practice. It should be noted that the dosing regimen in this real-world clinical setting did not strictly follow international guideline recommendations for triptan use. Specifically, some patients were prescribed zolmitriptan on a twice-daily basis rather than strictly on-demand during attacks, and dose escalation up to 15 mg/day was observed in some patients, which exceeds the recommended maximum daily dose of 10 mg. These prescribing practices reflect the real-world clinical context at our institution during the study period, but they are not presented as a recommended or guideline-concordant treatment protocol.

Regarding monthly consumption, the average number of zolmitriptan use days per month increased from baseline to 6 months (baseline: approximately 8–10 days/month; 6 months: approximately 18–22 days/month). This progressive increase in medication use raises concerns about potential medication overuse headache (MOH) development, which may have contributed to the limited efficacy observed in Group C. The absence of a defined maximum monthly treatment day limit is a significant limitation of this treatment regimen. Patients were also advised to avoid maintaining a forward neck stretch (more than 30 min of continuous flexion) and to engage in strengthening exercises for the cervical and neck area muscles.

#### 2.2.2 Continuous radiofrequency treatment of the C2 dorsal root ganglion under ultrasound guidance

After completing the preoperative assessment, the patient’s occipital area was prepped for surgery, extending to the nuchal line. It should be clarified that this procedure was administered as a routine therapeutic intervention rather than an acute treatment for migraine attacks, and it was performed under local anesthesia without the need for general anesthesia. In the operating room, an IV line was placed in the upper limb, and continuous monitoring of heart rate, blood pressure, and SpO2 was initiated. The patient was positioned in a lateral decubitus posture at 90°, with a thin pillow under the neck and the affected side facing upward. The surgical area was disinfected and draped aseptically. A low-frequency ultrasound probe (Shenzhen Wisonic Medical Technology Co., Ltd., model Labat SP, 3–5 MHz), coated with coupling gel and covered with a sterile sheath, was placed at the lower edge of the mastoid. It was then aligned longitudinally toward the atlantoaxial joint (Figures 2A,C), displaying the characteristic “stage light” image pattern. The probe was then rotated to an oblique axial position to simultaneously visualize the C2 dorsal root ganglion (Figures 2B,D), the spinal canal—which appears as a large oval hypoechoic structure—and the spinal cord, a smaller oval hypoechoic structure, creating the distinctive “turtle sign” image (16). Subsequently, color Doppler flow imaging (CDFI) was used to determine the spatial relationship between the C2 dorsal root ganglion, the vertebral artery, and nearby blood vessels, aiding in planning a safe puncture path (Figures 2E,F). Local infiltration anesthesia was administered with 0.5% lidocaine (2 mL). A radiofrequency needle (Boston Scientific; batch No. 32007160; specifications: 22G, 10 cm, 5 mm) was then advanced toward the C2 dorsal root ganglion. After removing the needle core, negative aspiration was performed to confirm there was no blood or cerebrospinal fluid return. A radiofrequency electrode (COSMAN MEDICAL, INC., United States; model RFG-4) was inserted. Sensory testing (50 Hz) and motor testing (2 Hz) were carried out at stimulation levels below 1.0 V to verify accurate needle placement. Once the electrode position was confirmed, the device was set to the “continuous radiofrequency mode.” Based on the patient’s intraoperative feedback indicating pain reproduction (such as electric shock, numbness, or pain in the ipsilateral temporal region, similar to preoperative sensations), radiofrequency ablation was carried out in three consecutive temperature stages: 42 °C, 45 °C, and 50 °C. Each stage lasted 90 s. Unlike traditional radiofrequency ablation, which typically uses temperatures ranging from 70 °C to 80 °C, this stepwise low-temperature protocol was adopted to minimize tissue damage and reduce the risk of complications. Afterward, the puncture needle was removed, and a sterile dressing was placed on the puncture site. Postoperative guidelines advised avoiding prolonged neck flexion (no more than 30 min of continuous flexion) and included regular neck and cervical muscle strengthening exercises. All procedures were carried out by the same physician, who had over 2 years of experience with this technique. For patients in Group R, non-steroidal anti-inflammatory drugs (loxoprofen sodium 60 mg per dose, maximum 180 mg/day) were permitted as a rescue analgesics for breakthrough headache attacks during the follow-up period. Patients were instructed to use rescue medication only when NRS pain intensity reached ≥ 4, and all rescue medication use was recorded in the electronic medical record system.

**Figure 2:**
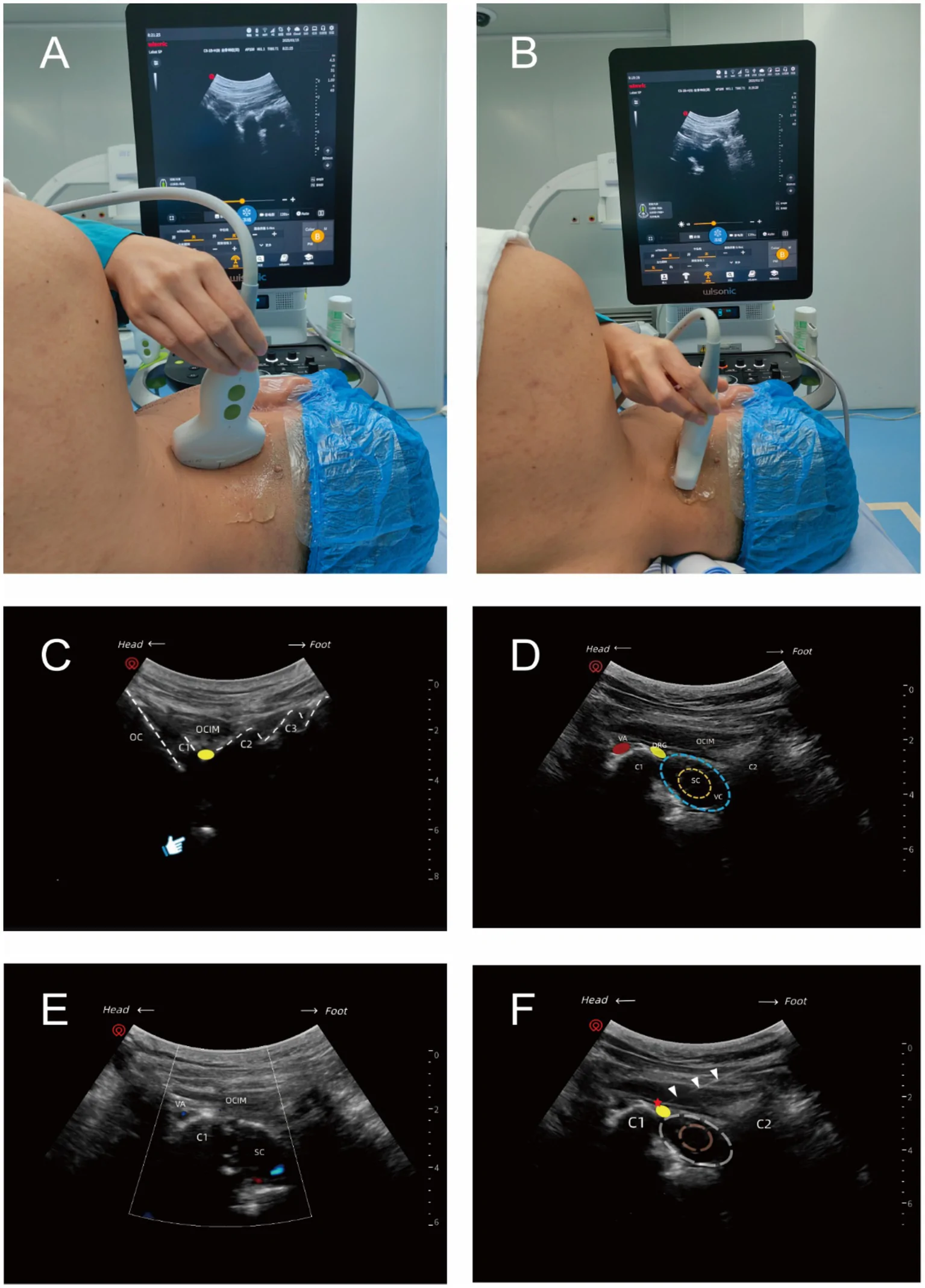
Ultrasound-guided imaging landmarks and puncture pathway for C2 dorsal root ganglion continuous radiofrequency treatment. Panel **(A)** shows the ultrasound probe positioned at the lower margin of the mastoid, performing longitudinal scanning of the atlantoaxial joint. Panel **(B)** presents the corresponding ultrasound image of **(A)**: Labels C1, C2, and C3 denote the first, second, and third cervical vertebrae, respectively; the yellow oval highlights the C2 dorsal root ganglion, and the finger points to its characteristic “stage light” imaging feature. Panel **(C)** depicts the ultrasound probe placed along the long axis of the obliquus capitis inferior muscle. Panel **(D)** displays the corresponding ultrasound image of **(C)**: the red oval indicates the vertebral artery, the yellow oval marks the C2 dorsal root ganglion, the large blue dashed oval represents the spinal canal, and the small light yellow dashed oval denotes the spinal cord. Panel **(E)** shows a long-axis ultrasound image of the obliquus capitis inferior muscle with color Doppler flow imaging (CDFI), which is used to evaluate the blood flow around the C2 dorsal root ganglion. Panel **(F)** illustrates the puncture pathway marked by a white triangle, and the red pentagram indicates the tip of the puncture needle.

### 2.3 Observation indicators

Pain Numerical Rating Scale (NRS) scores, attack frequency (times/month), attack duration (hours/episode), and zolmitriptan dosage (mg) were documented prior to treatment (T0) and at 1 week (T1), 1 month (T2), 3 months (T3), and 6 months (T4) following treatment. At 6 months post-treatment, therapeutic efficacy was assessed based on the NRS score improvement rate. Improvement rate was calculated as: [(Pre-treatment NRS score − Post-treatment NRS score)/Pre-treatment NRS score] × 100%. The efficacy grades were categorized as follows:

- Excellent: Improvement rate ≥ 75%
- Good response: 50% ≤ Improvement rate < 75%
- Fair response: 25% ≤ Improvement rate < 50%
- Poor response: Improvement rate < 25%

Questionnaires were used to assess the Hamilton Anxiety Scale (HAMA) (17), Hamilton Depression Scale (HAMD) (18), and Migraine Disability Assessment (MIDAS) questionnaire (19) before and after treatment.

#### 2.3.1 HAMA scoring criteria

A total score of 29 or higher indicates possible severe anxiety; a score between 21 and 28 indicates definite and marked anxiety; a score between 14 and 20 indicates definite anxiety; a score between 7 and 13 suggests possible anxiety; and a total score below 7 indicates no anxiety symptoms. Patients with HAMA scores of 14 or higher are considered to have anxiety.

#### 2.3.2 HAMD scoring criteria

A total score less than 8 indicates a normal state; 8 < total score < 20 indicates possible depression; 20 < total score < 35 indicates definite depression; and a total score of 35 or higher indicates severe depression. Patients with HAMD scores of 20 or above are considered to have depression.

#### 2.3.3 MIDAS evaluation

The MIDAS questionnaire includes five items that measure the impact of headache over the past 3 months: (1) Days missed from work or school due to headache; (2) Days with more than 50% reduction in productivity at work or school; (3) Days unable to perform household chores due to headache; (4) Days with more than 50% reduction in household work efficiency; (5) Days missed from family or social activities, such as visiting relatives, attending gatherings, or engaging in leisure activities like watching television or playing cards. The total of these five items forms the MIDAS score, which is classified into four categories based on severity: 0–5: Grade I (mild disability); 6–10: Grade II (mild to moderate disability); 11–20: Grade III (moderate disability); 21 or more: Grade IV (severe disability). A MIDAS score of 6 or higher indicates functional disability.

#### 2.3.4 Adverse reactions

The occurrence of adverse events such as drug allergy, puncture site pain, hematoma, infection, sensory or motor dysfunction, epidural hematoma, and local anesthetic toxicity was recorded.

### 2.4 Statistical analysis

Statistical analysis was conducted using GraphPad Prism version 10.0 (GraphPad Software Inc., San Diego, CA). Measurement data following a normal distribution are presented as mean ± standard deviation (±s), while those not conforming to a normal distribution are presented as median (interquartile range) [*M* (IQR)]. Independent sample *t*-tests were used for between-group comparisons of normally distributed data, and Wilcoxon rank-sum tests were employed for non-normally distributed data. Two-factor repeated-measures analysis of variance (ANOVA) was used to evaluate the main effects of time (pre- vs. post-treatment), group (Group C vs. Group R), and their interaction (time × group) on normally distributed outcomes (e.g., NRS scores). When a significant main effect or interaction was detected, *post hoc* pairwise comparisons (Bonferroni correction) were performed to analyze differences between each post-treatment time point and baseline (T0), as well as between groups at each time point.

Measurement data that do not follow a normal distribution were presented as median (interquartile range) (*M* [IQR]). Between-group comparisons were conducted using the Mann–Whitney test, while intra-group comparisons across different time points were analyzed with the Friedman test. Categorical data were expressed as percentages (%) and analyzed by the chi-square test. A *p*-value less than 0.05 was considered statistically significant.

## 3 Results

### 3.1 Comparison of general data

Initially, 69 patients scheduled for MRM were considered for this study. They were categorized based on treatment method into a conventional medication group (Group C, *n* = 34) and a continuous radiofrequency treatment group guided by ultrasound (Group R, *n* = 35).

No statistically significant differences were observed between the two groups regarding gender, age (Group C: 36 ± 6 years; Group R: 36 ± 6 years), headache side (left, right, or bilateral), body mass index (BMI, Group C: 21.8 ± 1.4 kg/m^2^; Group R: 22.4 ± 2.3 kg/m^2^), disease duration (Group C: 4.1 ± 1.3 years; Group R: 3.9 ± 1.6 years), or pre-treatment Numerical Rating Scale (NRS) scores (Group C: 7.5 ± 0.8; Group R: 7.5 ± 0.9) (all *p* > 0.05). These findings suggest that the baseline characteristics of both groups were similar (see Table 1).

**Table 1:** Comparison of baseline characteristics between the two groups (*n* = 69).

Group	Age (±s, years)	Headache side (n)			BMI (±s, kg/m^2^)	Disease duration (years, ±s)	Preoperative NRS (±s)
		Left	Right	Bilateral			
Group C	36 ± 6	8	10	16	21.8 ± 1.4	4.1 ± 1.3	7.5 ± 0.8
Group R	36 ± 6	11	10	14	22.4 ± 2.3	3.9 ± 1.6	7.5 ± 0.9
*t/χ* ^2^	0.055	0.593			−1.285	0.567	0.217
*P*	0.956	0.744			0.203	0.573	0.829

### 3.2 Comparison of pain NRS scores at different time points before and after treatment

Two-factor repeated-measures ANOVA revealed significant main effects of time (*F* = 589.76, *p* < 0.001), group (*F* = 428.31, *p* < 0.001), and time × group interaction (*F* = 196.54, *p* < 0.001) for NRS pain scores. Post-hoc pairwise comparisons with Bonferroni correction showed that, in both groups, NRS pain scores at all post-treatment time points (T1–T4) were significantly lower than pre-treatment scores (T0) in both groups (*p* < 0.001). Nonetheless, the degree of pain relief was notably greater in Group R compared to Group C. At T4 (6 months post-treatment), Group R’s NRS score was 3.60 ± 0.69, significantly lower than Group C’s 6.00 ± 0.55. This advantage for Group R remained consistent across all time points from T1 to T4 (all *p* < 0.001) (Table 2).

**Table 2:** Comparison of pain NRS scores at different time points (*x̄* ± s).

Group	T_0_	T_1_	T_2_	T_3_	T_4_	*F*	*P*
Group C (*n* = 34)	7.50 ± 0.79	4.82 ± 0.80a	5.5 ± 0.62	5.91 ± 0.51	6.00 ± 0.55	144.04	<0.001
Group R (*n* = 35)	7.46 ± 0.85	2.29 ± 0.62	2.71 ± 0.52	3.17 ± 0.38	3.60 ± 0.69	726.024	<0.001
*t*	−0.217	−14.776	−20.304	−25.054	−15.931		
*P*	0.829	<0.001	<0.001	<0.001	<0.001		

Compared with T_0_, *p* < 0.05. NRS = Numerical Rating Scale.

### 3.3 Comparison of headache attack frequency before and after treatment

Starting 1 month after treatment (T2), the frequency of headache attacks (times per month) decreased significantly compared to pre-treatment (T0) in both groups (*p* < 0.001). The reduction was notably greater in Group R. At 6 months post-treatment (T4), Group R’s average attack frequency was 3.20 ± 1.83 times per month, significantly lower than 5.32 ± 2.31 in Group C. The differences between the groups at T2 and T3 were also statistically significant (*p* < 0.001) (Table 3).

**Table 3:** Comparison of headache attack frequency (times/month, *x̄*±s).

Group	T_0_	T_2_	T_3_	T_4_	*F*	*P*
Group C (*n* = 34)	6.79 ± 5.39	4.29 ± 2.05	4.88 ± 2.51	5.32 ± 2.31	20.95	<0.001
Group R (*n* = 35)	6.60 ± 4.75	2.26 ± 1.15	2.57 ± 1.36	3.20 ± 1.83	35.661	<0.001
*t*	0.159	5.069	4.741	4.232		
*P*	0.874	<0.001	<0.001	<0.001		

Compared with T_0_, *p* < 0.05.

### 3.4 Comparison of headache attack duration before and after treatment

At 1 month (T2), 3 months (T3), and 6 months (T4) post-treatment, the average length of a single headache episode (hours per attack) was notably shorter than at baseline (T0) in both groups (*p* < 0.001). The duration in Group R remained consistently shorter than in Group C. For instance, at T4, the median attack duration in Group R was 4.00 h, compared to 8.00 h in Group C. The differences between the groups at T2 and T3 were also statistically significant (*p* < 0.001) (Table 4).

**Table 4:** Comparison of headache attack duration (hours/episode, *M* [P25, P75]).

Group	T_0_	T_2_	T_3_	T_4_	*F*	*P*
Group C (*n* = 34)	10.00 (6.00, 24.00)	6.00 (4.00, 15.00)	8.00 (4.25, 19.50)	8.00 (5.25, 20.00)	92.245	<0.001
Group R (*n* = 35)	10.00 (6.00, 24.00)	3.00 (2.00, 4.00)	4.00 (2.50, 6.00)	4.00 (3.00, 6.00)	82.27	<0.001
*Z*	−0.157	−3.665	−3.319	−3.702		
*P*	0.875	<0.001	<0.001	<0.001		

Compared with T_0_, *p* < 0.05.

### 3.5 Comparison of modified MacNab clinical efficacy (based on NRS improvement rate) at 6 months after treatment

Six months after treatment, the modified MacNab criteria were used to assess therapeutic effectiveness. The overall effective rate (including excellent, good, and fair outcomes) was notably higher in Group R compared to Group C (88.57% vs. 35.29%). Specifically, in Group R, 9 patients (25.71%) achieved an ‘excellent’ rating, and 13 patients (37.14%) achieved a ‘good’ rating. Conversely, in Group C, only 2 patients (5.88%) were rated as “excellent,” and 3 patients (8.82%) as “good.” The difference between the groups was statistically significant (*χ*^2^ = 23.407, *p* < 0.001) (see Table 5).

**Table 5:** Comparison of Modified MacNab Clinical Efficacy at 6 months after treatment [*n* (%)].

Group	Excellent	Good	Fair	Poor	Total effective rate (%)
Group C (*n* = 34)	2 (5.88)	3 (8.82)	7 (20.59)	22 (64.71)	12 (35.29)
Group R (*n* = 35)	9 (25.71)	13 (37.14)	9 (25.71)	4 (11.42)	31 (88.57)
*χ* ^2^					23.407
*P*					<0.001

### 3.6 Comparison of VRS, HAMA, and HAMD scores before and after treatment

At all post-treatment time points (T1–T4), both groups exhibited significant reductions in VRS scores for nausea and vomiting, as well as in HAMA and HAMD scores, compared to pre-treatment (T0) (*p* < 0.001). However, the improvements were more noticeable in Group R.

At T4, the median VRS score for Group R was 0.00 (interquartile range: 0.00–1.00), significantly lower than Group C’s 3.00 (interquartile range: 2.00–3.00). The average HAMA score in Group R was 3.94 ± 1.06, compared to 8.74 ± 2.14 in Group C. The mean HAMD score in Group R was 5.29 ± 0.93, while in Group C it was 11.76 ± 3.62. All these parameters showed statistically significant differences between groups at T1–T4 (*p* < 0.001) (see Table 6).

**Table 6:** Comparison of VRS, HAMA, and HAMD scores at different time points (*M* [P25, P75], *x̄* ± s).

Evaluation index	Group	T_0_	T_1_	T_2_	T_3_	T_4_	*F*	*P*
VRS	Group C (*n* = 34)	3.00 (3.00, 3.00)	2.00 (2.00, 2.00)	2.00 (2.00, 2.00)	2.00 (2.00, 3.00)	3.00 (2.00, 3.00)	121.098	<0.001
	Group R (n = 35)	3.00 (3.00, 3.00)	0.00 (0.00, 0.00)a	0.00 (0.00, 0.00)a	0.00 (0.00, 0.00)a	0.00 (0.00, 1.00)a	82.293	<0.001
	*Z*	−0.142	−7.216	−7.127	−7.09	−7.124		
	*F*	0.887	<0.001	<0.001	<0.001	<0.001		
HAMA	Group C (*n* = 34)	11.24 ± 2.93	10.32 ± 2.73	9.53 ± 2.44	9.24 ± 2.40	8.74 ± 2.14	51.31	<0.001
	Group R (*n* = 35)	11.34 ± 3.06	6.11 ± 1.21	4.89 ± 1.08	4.46 ± 0.92	3.94 ± 1.06	226.647	<0.001
	*t*	−0.149	8.249	10.174	10.861	11.865		
	*P*	0.882	<0.001	<0.001	<0.001	<0.001		
HAMD	Group C (*n* = 34)	13.97 ± 4.50	12.65 ± 4.17	12.21 ± 4.04	12.00 ± 3.83	11.76 ± 3.62	12.791	<0.001
	Group R (*n* = 35)	14.89 ± 5.19	7.43 ± 0.50	6.54 ± 0.51	5.83 ± 0.66	5.29 ± 0.93	121.922	<0.001
	*t*	−0.781	7.247	8.12	9.262	10.123		
	*P*	0.438	<0.001	<0.001	<0.001	<0.001		

Compared with T0, *p* < 0.05.

### 3.7 Comparison of MIDAS scores before and after treatment

At 3 months (T3) and 6 months (T4) following treatment, both groups showed a significant decrease in MIDAS Questionnaire scores compared to pre-treatment levels (T0) (*p* < 0.001). The reduction was notably greater in Group R than in Group C. At T4, Group R’s MIDAS score was 9.89 ± 6.00, considerably lower than Group C’s 43.94 ± 13.73. The difference between groups at T3 was also statistically significant (*p* < 0.001) (see Table 7).

**Table 7:** Comparison of MIDAS scores between the two groups (*x̄* ± s).

Group	T0	T3	T4	*F*	*P*
Group C (*n* = 34)	58.68 ± 13.98	30.56 ± 12.48	43.94 ± 13.73	42.501	<0.001
Group R (*n* = 35)	54.51 ± 13.91	8.29 ± 3.79	9.89 ± 6.00	547.306	<0.001
*t*	−1.24	−9.968	−13.285		
*P*	0.219	<0.001	<0.001		

Compared with T0, *p* < 0.05.

### 3.8 Incidence of adverse reactions and complications after treatment

In Group R, only two patients had mild scalp numbness after surgery, which resolved on its own within 7 days. No serious adverse reactions or complications, such as drug allergy, puncture site hematoma or infection, sensory or motor dysfunction, epidural hematoma, or local anesthetic toxicity, were observed in either group. The low complication rate in Group R may be attributed to the stepwise low-temperature ablation protocol (42 °C → 45 °C → 50 °C) adopted in this study, which avoids the tissue damage associated with high-temperature radiofrequency ablation (70–80 °C) used in traditional procedures.

**Table 8:** Comparison of zolmitriptan dosage in Group C at different time points (*x̄* ± s, mg/day).

Time point	Zolmitriptan dosage (mg/day)
T0 (Baseline)	5.00 ± 0.00
T1 (1 week)	7.88 ± 2.15a
T2 (1 month)	10.65 ± 2.83a
T3 (3 months)	12.35 ± 2.51a
T4 (6 months)	13.18 ± 1.89a
*F*	112.63
*P*	< 0.001

Compared with T0, *p* < 0.05.

### 3.9 Comparison of zolmitriptan dosage in Group C at different time points

Since zolmitriptan was only administered in Group C (conventional medication group) and not in Group R (radiofrequency treatment group), the dosage of zolmitriptan was analyzed exclusively in Group C. Zolmitriptan dosage (mg/day) was recorded at baseline (T0) and at 1 week (T1), 1 month (T2), 3 months (T3), and 6 months (T4) post-treatment. The results showed that the average zolmitriptan dosage in Group C increased gradually over the follow-up period. At T0, the mean daily dosage was 6.82 ± 0.39 mg (fixed initial dose: 2.5 mg twice daily). Due to insufficient symptom relief, the dosage was adjusted in 28 patients (82.35%) during the treatment period. At T1, T2, T3, and T4, the mean daily dosages were 0.91 ± 0.67 mg, 4.27 ± 0.71 mg, 4.82 ± 0.58 mg, and 5.35 ± 0.85 mg, respectively. Repeated-measures analysis of variance (ANOVA) revealed a statistically significant difference in zolmitriptan dosage among different time points (*F* = 487.961, *p* < 0.001). *Post hoc* pairwise comparisons indicated that the dosage at each post-treatment time point (T1–T4) was significantly higher than that at baseline (T0) (all *p* < 0.001), and the dosage continued to increase with prolonged treatment duration.

Notably, despite the progressive increase in zolmitriptan dosage in Group C, the improvements in headache symptoms (NRS score, attack frequency, duration), psychological status (HAMA, HAMD scores), and functional disability (MIDAS score) were still significantly inferior to those in Group R (all *p* < 0.001). This progressive dose escalation with diminishing returns is consistent with the development of medication overuse headache (MOH), a well-recognized complication of excessive triptan use. The observed worsening of symptoms despite dose increase likely reflects MOH and/or tachyphylaxis rather than intrinsic limitations of appropriately prescribed pharmacological therapy (Table 8).

### 3.10 Comparison of rescue analgesic consumption patterns

The frequency of rescue analgesic (loxoprofen sodium) use in Group R was analyzed before and after CRF treatment. At baseline (T0), the mean frequency of rescue medication use was 6.8 ± 1.23 days per month. Following CRF treatment, rescue medication use decreased significantly at all follow-up time points: T1 (1 week): 0.26 ± 0.44 days/month; T2 (1 month): 1.37 ± 0.6 days/month; T3 (3 months): 1.97 ± 0.82 days/month; T4 (6 months): 2.80 ± 0.83 days/month. Repeated-measures ANOVA showed a significant reduction in rescue medication use over time (*F* = 401.406, *p* < 0.001), with post-hoc comparisons confirming significant reductions at all post-treatment time points compared with baseline (all *p* < 0.001). This reduction in rescue analgesic consumption further supports the therapeutic efficacy of CRF treatment.

## 4 Discussion

The present study compared the therapeutic effects of ultrasound-guided continuous radiofrequency treatment of the C2 dorsal root ganglion (Group R) with conventional zolmitriptan medication (Group C) over a 6-month follow-up. The pre-post study design used in the CRF arm of our study is consistent with recent literature evaluating interventional headache treatments. For example, a recent study using a similar pre-post design demonstrated significant improvements with occipital nerve stimulation in chronic migraine patients (12) supporting the validity of this approach for evaluating neuromodulatory interventions. The results showed that both treatments could reduce headache symptoms and related problems in patients with MRM. These results are consistent with the findings of Lionetto et al. (10) and Mercieri et al. (13). However, the effectiveness of Group R was significantly better. From 1 week to 6 months after treatment (T1–T4), Group R experienced greater decreases in pain NRS scores and scores on the anxiety and depression scales (HAMA and HAMD). Between 1 and 6 months after treatment (T2–T4), Group R also demonstrated better improvements in headache attack frequency and duration. Between 3 and 6 months (T3–T4), they showed greater improvement in MIDAS scores. At 6 months post-treatment, the modified MacNab total effective rate in Group R (88.57%) was notably higher than that in Group C (35.29%). Only two cases of mild, transient scalp numbness occurred in Group R, with no severe adverse reactions observed, indicating good safety.

A notable observation from this study is that headache symptoms during the non-menstrual period in MRM patients showed significant improvements following C2 DRG CRF ablation—as evidenced by reduced NRS pain scores, decreased attack frequency and duration, and alleviated nausea/vomiting (see Sections 3.2–3.4, 3.6). This exploratory finding preliminarily suggests a potential link between non-menstrual headaches in MRM and cervicogenic factors. Traditional theories primarily emphasize the role of hormonal fluctuations (e.g., changes in estrogen and progesterone levels) during the menstrual cycle in the pathogenesis of MRM (20), while the underlying causes of headaches during the non-menstrual period have been less frequently explored. Cervicogenic headache stems from abnormal changes in the cervical spine or soft tissues of the neck (21), and its pain distribution commonly overlaps with that of MRM, especially in the occipital and temporal areas. Our clinical observations and study results suggest that the C2 dorsal root ganglion could be a significant factor throughout all stages of MRM, particularly during the non-menstrual phase, where cervicogenic factors may act as key triggers for headache attacks. This is further supported by the imaging findings of cervical curvature changes in some MRM patients enrolled in this study, which may be associated with C2 DRG irritation or dysfunction. This finding offers a new perspective for further exploring MRM pathophysiology. Future research may examine the intrinsic link between the C2 dorsal root ganglion and non-menstrual headaches in MRM from neuroanatomical and neurophysiological viewpoints, thereby providing stronger theoretical support for understanding the disease’s development in MRM.

During the menstrual cycle, hormonal fluctuations (e.g., changes in estrogen and progesterone levels) modulate the release and metabolism of neurotransmitters such as serotonin and calcitonin gene-related peptide (CGRP) (20), which directly impairs neuronal membrane stability by altering the activity of ion channels (e.g., sodium and calcium channels) and enhances neural sensitivity (22).

For MRM patients with underlying cervicogenic factors, the C2 dorsal root ganglion (C2 DRG) is often in a pre-existing hypersensitive state—likely due to cervical curvature abnormalities or soft tissue irritation (21)—making it more vulnerable to hormonal changes during menstruation. This synergistic effect further amplifies neuronal excitability and lowers the threshold for headache initiation (23).

Notably, the molecular mechanisms underlying the therapeutic effects of zolmitriptan and ultrasound-guided C2 DRG continuous radiofrequency (CRF) differ substantially. Zolmitriptan exerts acute analgesic effects by targeting 5-hydroxytryptamine 1B/1D receptors to inhibit CGRP release from trigeminal nerve endings and constrict dilated intracranial blood vessels (24). However, this pathway is highly dependent on endogenous neurotransmitter levels, leading to potential efficacy attenuation with long-term use, and it fails to address the intrinsic functional abnormalities of the C2 DRG. In contrast, the stepwise low-temperature CRF protocol (42 °C → 45 °C → 50 °C) adopted in this study—distinct from traditional high-temperature radiofrequency (70–80 °C) (25, 26)—exerts therapeutic effects through three key molecular pathways: (1) Regulation of sodium channels: Radiofrequency energy downregulates the expression of Nav1.8 sodium channels in C2 DRG neurons, a critical molecule for pain signal generation and conduction, thereby directly inhibiting the initiation and transmission of nociceptive signals (27); (2) Improvement of the inflammatory microenvironment: CRF reduces the local release of pro-inflammatory factors such as CGRP (28), modulates the perineural microenvironment, and enhances cervical blood circulation to reduce neural excitability (29); (3) Inhibition of neural conduction: Moderate thermal modulation of C2 DRG function blocks the upward transmission of pain signals to the central nervous system, cutting off the migraine pain pathway at its source (28).

Consistent with previous studies on cervicogenic headache (14, 15), our findings confirm that targeting the C2 DRG via CRF can effectively alleviate MRM symptoms, particularly non-menstrual headaches. This is supported by He et al.’s (16) observation that ultrasound-guided C2 DRG access enables precise modulation of neural activity, while Chazen et al. (29) reported similar efficacy of C2 DRG radiofrequency in reducing headache-related hypersensitivity. Thus, the greater observed clinical improvements with CRF relative to zolmitriptan in this study may be attributed to its ability to target the underlying molecular abnormalities of C2 DRG hypersensitivity, rather than merely providing symptomatic relief. These results provide preliminary evidence that cervicogenic factors, mediated by specific molecular pathways, contribute to MRM pathogenesis—especially during the non-menstrual phase.

The results also showed that, compared to conventional zolmitriptan therapy, continuous radiofrequency treatment led to greater improvements in nausea and vomiting related to headaches. At 6 months after treatment, the VRS score for nausea and vomiting in Group R was 0.00 (0.00, 1.00), versus 3.00 (2.00, 3.00) in Group C. This difference may be explained by two mechanisms: (1) continuous radiofrequency treatment of the C2 dorsal root ganglion can adjust neural conduction, block or reduce abnormal nerve impulses, and thus decrease vagal stimulation and alleviate nausea and vomiting (30); (2) continuous radiofrequency treatment can reduce neck muscle tension, relieve vascular compression caused by the muscles, and enhance cervical blood circulation (31).

Furthermore, ongoing radiofrequency treatment not only alleviated headache symptoms but also markedly improved anxiety, depression, and migraine-related disability in patients. MRM patients with chronic headaches often face negative emotions like anxiety and depression, which can worsen headache symptoms and increase migraine-related disability, creating a vicious cycle. Continuous radiofrequency treatment effectively relieves headaches (29), breaks this vicious cycle, and enhances patients’ psychological well-being (6). In contrast, while conventional medication offered only partial headache relief, it was less effective in reducing anxiety, depression, and migraine disability compared to continuous radiofrequency treatment of the C2 dorsal root ganglion. This highlights the importance of addressing both headache symptoms and psychological states in MRM management. Ultrasound-guided continuous radiofrequency of the C2 dorsal root ganglion shows a clear advantage in achieving this dual therapeutic goal (alleviating headache symptoms and improving mental health).

An important limitation of the pharmacological arm of this study is that the zolmitriptan dosing regimen did not conform to current international guidelines. Triptans are recommended for acute abortive treatment only, to be taken at the onset of migraine attacks, with a maximum daily dose of 10 mg and a maximum of 10 treatment days per month to minimize the risk of medication overuse headache (MOH). The prescribing practices observed in this study—including twice-daily dosing and dose escalation up to 15 mg/day—are not guideline-concordant and likely contributed to the progressive loss of efficacy and potential MOH development observed in Group C. Therefore, the between-group differences observed in this study should not be interpreted as evidence that CRF is superior to properly administered zolmitriptan therapy; rather, they reflect the outcomes of a suboptimal pharmacological regimen compared with a neuromodulatory intervention in a real-world clinical setting.

This study has several limitations that should be acknowledged. First, as a retrospective study, it is inherently susceptible to selection bias, recall bias, and confounding factors. Selection bias may have arisen from non-random treatment allocation based on patient preference, clinical judgment, and contraindications. Recall bias is partially mitigated by the use of a standardized electronic medical record system with prospectively recorded data, but cannot be entirely eliminated. These limitations may affect the internal validity and generalizability of the results. Second, the sample size was relatively small (*n* = 69), which limits the statistical power to detect smaller effect sizes and reduces the generalizability of the findings. Although MRM is highly prevalent in the general population, the strict inclusion criteria (e.g., regular menstrual cycles, no preventive medication in the past 3 months, NRS ≥ 4) and the single-center design limited the number of eligible patients. Future multicenter studies with larger sample sizes are needed to confirm these findings. Fourth, the study compares two different treatment modalities (a neuromodulatory procedure vs. pharmacological therapy) that serve different clinical purposes, which limits the direct interpretability of between-group differences. The comparison reflects real-world clinical practice and cannot support definitive conclusions regarding relative therapeutic performance. Fifth, the efficacy of C2 DRG CRF for migraine has not been established through adequately powered randomized controlled trials. The current evidence base consists primarily of case reports and small observational studies. This study provides additional observational data but should be considered exploratory and hypothesis-generating rather than confirmatory. Well-designed randomized controlled trials are needed to establish the efficacy and safety of this intervention for MRM. Additionally, basic research at the molecular and cellular levels is required to clarify the exact mechanism by which C2 DRG CRF ablation alleviates MRM symptoms. Future research should include large-sample, multicenter, prospective randomized controlled trials to further verify the long-term efficacy and safety of ultrasound-guided continuous radiofrequency treatment of the C2 dorsal root ganglion for MRM. Additionally, the mechanisms behind this treatment are not yet fully understood. More basic research, especially at the molecular and cellular levels, is necessary to clarify its mechanism of action.

## 5 Conclusion

In conclusion, ultrasound-guided continuous radiofrequency treatment of the C2 dorsal root ganglion was associated with greater improvements in headache symptoms and psychological status compared with zolmitriptan therapy in this retrospective observational study. These preliminary findings should be interpreted with caution given the different treatment modalities and study design limitations. Furthermore, this study suggests that non-menstrual headaches in MRM may be associated with cervicogenic factors, providing a new perspective and direction for the clinical management of MRM. However, further research is needed to optimize the treatment protocol and clarify the underlying mechanisms.

Additionally, this study provides preliminary evidence suggesting that non-menstrual headaches in MRM may be linked to cervicogenic factors, providing a new perspective and direction for MRM treatment. However, further research is needed to optimize the treatment protocol and better understand the underlying mechanisms.

## Data Availability

The raw data supporting the conclusions of this article will be made available by the authors, without undue reservation.
